# Supplementary material for: Associated factors of pelvic organ prolapse among patients at Public Hospitals of Southern Ethiopia: A case-control study design
Source: PLoS One. 2023 Jan 18;18(1):e0278461. doi: 10.1371/journal.pone.0278461 (PMC9847956; doi:10.1371/journal.pone.0278461)
Supplement: S1 File — (PDF) [file pone.0278461.s001.pdf]

Supporting information

S1 File: Confidentiality and informed consent statement

My name is _________I am from__________. I am part of a team of people who are carrying out study on *predictors of pelvic organ prolapse and reasons associated with delayed treatments among patients at selected referral and general hospitals of Southern Ethiopia* by Asfaw Borsamo who is attending his Master's degree in Human Anatomy at University of Gondar. I brought these questions to you in order to find out conditions regarding to youth friendly service utilization and associated factors among preparatory school students in North Shewa zone. The questionnaire will take about 30-40 minutes to fill. The purpose of this study is to get more information on factors contributing to pelvic organ prolapse and reasons for delayed treatments. Therefore, your honest and genuine involvement by responding to the questions prepared is highly appreciated and helpful to attain the objective of the study. Your name will not be written on this form and no individual response will be reported to anybody. Hence, your answers are completely confidential. If you do not have answer for any question or you do not want to respond, you can withdraw the interview at any time. I would greatly appreciate your help in participating in this study. Would you be willing to participate?

If yes, let us proceed by signing this document.

If no, thank you and stop here.

Participant's signature ____________________ Date______________

Data collector's signature___________________ Date ____________

S2 File: English version data collection checklist

This interviewer-guided questionnaire was developed aiming to assess factor associated with pelvic organ prolapse and reasons associated with delayed treatments.

Ref. No___________________________

S.No	**Part one: Objective findings**	
1	Is there POP?	Yes
		No
2	If yes, what is stages of POP?	
3	What is BMI (kg/m^2)?	
4	Delayed treatment	Yes
		No
	**Part two: Factors associated with POP**	
	a) ***Sociodemographic and economic***	
5	What is your age?	
6	What is your ethnicity?	Gurage
		Amhara
		Kambata
		Wolaita
		Hadiya
		Sidama
		Oromo
		Gamo

		Others
7	What is your religion?	Orthodox
		Protestants
		Muslim
		Others
8	What is your monthly income?	
9	What is your residency?	Rural
		Semi-urban
		Urban
10	What is your marital status?	Single
		Married
		Widowed
		Divorced
11	What is your educational status?	No schooling
		Primary school
		Secondary school
		Diploma and above
12	What is your occupational status?	House wife
		Farmer
		Other physical work
		Professionally employed
13	What was your age at marriage?	
14	Do you have family history of POP?	Yes

		No
	b) Obstetric factors	
15	How many times did you give birth (parity)?	
16	What was mode of delivery at last delivery?	Spontaneous Vaginal delivery
		Assisted Vaginal delivery
		CS delivery
17	Where did you give your last birth?	Health institution
		Home
18	Do you have history of home delivery?	Yes
		No
19	When did you return to work after delivery	Before 42 days
		After 42 days
20	How long is your delivery interval?	
21	What is duration of labor at your last delivery?	
22	Do you have history of duration of labor >24 hr?	Yes
		No
23	What was your age at first delivery?	
24	What was your age at last delivery?	
25	Dou you have history of Sphincter damage/ Vaginal tear	Yes
		No
26		Yes

	Do you have history of instrumental delivery?	No
	c) Factors that increase intra-abdominal pressures	
27	Do you have history of chronic cough(> 3 wk)?	Yes
		No
28	Do you have history of chronic constipation(>3 months)?	Yes
		No
29	Do you carry heavy objects?	Yes
		No

Reason that hinder early treatments

30	How long is the onset of prolapse?	
	**Why did you delay for treatments?**	
31	Because of you Lack of support?	Yes
		No
32	Low Income?	Yes
		No
33	Because lack of transportation?	Yes
		No
34	Because unavailability of treatment center?	Yes
		No
35	Because do you Believe it as normal?	Yes

		No
36	Because Fearing surgery?	Yes
		No
37	Because of Culture?	Yes
		No
38	Because of Afraid of losing social value/stigma?	Yes
		No
39	Because of Fear of disclosure?	Yes
		No
40	Because of busy?	Yes
		No

Thank you for your participation!!!

S3 File: Amharic version collection checklist

ክፍል አራት፡ ቃለ-መጠይቅ (በአማርኛ)

በጥናቱ ዙሪያ አጠቃላይ መረጃ ለመስጠትና ስምምነት ለመውሰድ የተዘጋጀ ቅጽ

በጎንደር ዩኒቨርሲቲ ጤና ሳይንስ ኮሌጅ በሰውነት አካላት ጥናት ትምህርት ክፍል መርሃ ግብር

የተከበሩ የጥናቱ ተሳታፊ!

በቅድሚያ እንደ ምን አደራቹሁ/ዋላቹህ?! እኔ---እባላለሁ።

በአሁኑ ስዓት በጎንደር ዩንቨርስቲ በህክምናና ጤና ሳይንስ ኮሌጅ በሰውነት አካላት ጥናት ትምህርት ክፍል

መርሃ ግብር የማስተርስ/ሁለተኛ ድግሪ ተመራቂ ተማሪ የሆኑት **አቶ አስፋው ቦርሳሞ** በደቡብ ክልል ባሉ ሆስፒታሎች የእናቶች መውለጃ ብልቶች ወደ ውጭ እንዲወጡ የሚያደርጉት ምክንያቶችን ምን እንደሆኑና እንዲሁም በጊዜ እንዳይታከሙ በሚከለክሉኣቸው ምክንያቶች ላይ ጥናት እንዲያደርግ ዩንቨርስቲው ወጭውን በመሸፈን ፈቅዶታል። ለዚህም ይረዳ ዘንድ መረጃ እንድሰበስብለት ከመረጣቸው ባለሙያዎች መካከል እኔ አንዱ ነኝ። ስለሆነም ከመሳተፍዎ በፊት ግን የጥናቱን ጠቅላላ ይዘት እና ዓላማ እንደሚከተለው ላብራራልዎት እወዳለሁ።

የጥናቱ ዓላማ፡- የዚህ ጥናት ዋና ዓላማ በደቡብ ክልል ባሉ ሆስፒታሎች የእናቶች መውለጃ ብልቶች ወደ ውጭ እንዲወጡ የሚያደርጉት ምክንያቶችን ምን እንደሆኑና እንዲሁም በጊዜ እንዳይታከሙ በሚከለክላቸው ምክንያቶች ምን እነደሆኑ ለማጥናት ነው።

ከጥናቱ በመሳተፍዎ የሚያገኙት ጥቅም እና ጉዳት

ጥቅም፡- በዚህ ጥናት በመሳተፉ ቀጥተኛ የሆነ ገንዘብም ሆነ ሌላ ጥቅም አያገኙም። ነገር ግን የእርስዎ በዚህ ጥናት መሳተፍ የእናቶች መውለጃ ብልቶች ወደ ውጭ እንዲወጡ በሚያደርጉት ምክንያቶችን ምን እንደሆኑና እንዲሁም በጊዜ እንዳይታከሙ በሚከለክላቸው ምክንያቶች ምን እንደሆኑ ለማወቅ ያስችላል። ከዚህ ጥናት ሚገኙ መረጃዎች በዚህ ችግር የሚጠቁ እናቶች ቁጥር ለመቀነስና እነዲሁም ተጠቂዎችን በጊዜ ህክምና እንዲያገኙ፤ መንግስት ችግሩን ዘለቄታ ባለው መንገድ እስቲራቴጂ ነድፎ እንድሰራ ለማድረግ ወሳኝነት አለው። በተጨማሪም ፣ ከዚህ ጥናት የሚገኙ መረጃዎች ችግሩን ለመግታት የሚመለከታቸውና ፍላጎት ያላቸው ግለሰችና ድርጅቶች እንድረባረቡ ያነሳሳል።

ጉዳት፡- በመጀመሪያ ጥናት የሚያካሂደው አካል ጥናቱን ለማካሄድ ከጎንደር ዩንቨርስቲ ህጋዊ ፍቃድ የተሰጠው ሲሆን የደቡብ ክልል ጤና ቢሮ ጥናቱን እንድሰራ ፈቅደውለታል። ከዚህ በተጨማሪ ጥናቱ

የሚካሄደው በቃለ መጠይቅ ነው። በአጠቃላይ ከጥናቱ በመሳተፍም ባለመሳተፍም በእስዎና በቤተሰብዎት ላይ የሚመጣው ምንም አይነት ችግር ወይም ጉዳት የለም፤ ቢበዛ 20 ደቂቃ ከመስጠት በሰተቀረ።

መብትን በተመለከተ፡ ጥናቱ በፍቃደኝነት ላይ የተመሰረተ ስለሆነ በጥናቱ ዉስጥ የመሳተፍም ያለመሳተፍም እንዲሁም የጥናቱ መጠይቅ ካልተመቸዎት በፈለጉት ጊዜ የማቋረጥ መብት አለዎት።

ሚስጢር መጠበቅን በተመለከተ፡ የእርስዎ ስም በመጠይቁ ወረቀት ላይ አይፃፍም። የሚትሠጡን መረጃ በምንም መልኩ ለሰስተኛ ወገን አይታይም።

ስለጥናቱ ማንኛውም ዓይነት ጥያቄ ቢኖርዎት ወይም ስለጥናቱ የመጨረሻ ውጤት ማወቅ ቢያስፈልግዎት በሚከተለው የዋናው ተመራማሪ አድራሻ ማግኘት ይችላሉ።

የተማራማሪዎች አድራሻ፡-

1. አስፋው ቦርሳሞ፡ ዋና ተመራማሪ ስልክ፡ 0984867398፣ ኢሜይል asеborsa@gmail.com
2. ያሬድ አስማረ-------በጎንደር ዩንቨርስቲ የተመራማሪው አማካሪ
3. አያናው ወርቁ-------በጎንደር ዩንቨርስቲ የተመራማሪው ረዳት አማካሪ
4. ሞሃመድ ኡመር----በጎንደር ዩንቨርስቲ የተመራማሪው ረዳት አማካሪ

በአጠቃለይ በጥናቱ ዙሪያ በተደረገለዎት ገለጻ ተስማምተውና አምነውበት ከሆነ በጥያቄው ለመሳተፍ ፍቃደኛ ኖት?

1. **አዎ**-----ጥያቄውን ይቀጥሉ
2. **አይደለውም**------ስለነበረን ቆይታ አመሰግናለው በማለት ጥያቄውን ያቋርጡ

የስምምነት ቅጽ ለወላጅ

እኔ ከዚህ በታች የፈረምኩት ግለሰብ በደቡብ ክልል ባሉ ሆስፒታሎች የእናቶች መውለጃ ብልቶች ወደ ውጭ እንዲወጡ የሚያደርጉት ምክንያቶችን ምን እንደሆኑና እንዲሁም በጊዜ እንዳይታከሙ በሚከለክሉኣቸው ምክንያቶችን ለማወቅ በሚደረገው ጥናት ያለውን ጥቅምና ጉዳት በመረዳት በፈቃደኝነት ላይ የተሳተፍኩ ሲሆን ከዚህ ቀጥሎ በሚገኘው መጠይቅ የምሰጠው መረጃም ሚስጢርነቱ የተጠበቀ እንደሚሆንና መጠይቁ ካልተመቸኝ በፈለኩት ስዓት የማቋረጥ መብት እንዳለኝ በሚገባ ተነግሮኛል፡፡ በጥናቱ ውስጥ ተሳታፊ መሆኔም አለመሆኔም በግል ሕይወቴ ውስጥ ችግር እንደማያመጣብኝም ተነግሮኛል፡፡ በመጨረሻም ስለ ጥናቱ እና የጥናቱ ተሳታፊ እንደመሆኔ ባለኝ መብት ዙሪያ ጥያቄ ቢኖረኝ አቶ አስፋው ቦርሣሞ የተባለውን የጥናቱ ዋና ባለቤት ማናገር አንደምችልም ተረድቻለሁ፡፡

የመረጃ ሰጭው መለያ ኮድ----------- ፊርማ-------------ቀን------------------

የመረጃ ሰብሳቢው ፊርማ----------------ቀን-------------------------------------

ቃለ-መጠይቅ

በደቡብ ክልል ባሉ ሆስፒታሎች የእናቶች መውለጃ ብልቶች ወደ ውጭ እንዲወጡ የሚያደርጉት ምክንያቶችን ምን እንደሆኑና እንዲሁም በጊዜ እንዳይታከሙ በሚከለክሉኣቸው ምክንያቶችን ለማወቅ የሚደረግ ጥናት

Ref. No___________________________

S.No	**ክፍል አንድ: በባለ-ሙያ ታይቶ የተገኙ ግኝቶች**	
1		አዎ

	የመውለጃ ብልቶች እየተሳቡ ወደ ውጭ ይወጣሉን?	አይደለም
2	የህመሙ ደረጃ ምን ያክል ነው?	
3	መጠነ-ውፈረትዋ ምን ያክል ነው (kg/m^2) (kg/m^2)?	
4	ለህክምና ዘገይተዋል?	አዎ
		አይደለም
	**ክፍል ሁለት: ህመሙ እንዲፈጠር የሚደርጉ ምክንቶች**	
	ሀ. ማህበራዊ መስተጋብር ምክንያ ቶች	
5	ዕድሜዎት ስንት ነው?	
6	ዘርዎት ምንድነው?	ጉራጌ
		አማራ
		ከንባታ
		ወላይታ
		ሀድያ
		ሲዳማ
		ኦሮሞ
		ጋሞ
		ሌሎችም

7	ኃይማትዎት ምንድነው?	ኦርቶዶክስ
		ፕሮተስትታንት
		ሙስሊም
		ሌሎችም
8	ወርዊ ገብዎት ስንት ነው?	
9	መኖሪያውት ዬት ነው?	ገጠር
		በከፊል ከተማ
		ከተማ
10	የጋብቻዎት ሁኔታስ?	ያላገባች
		ያገባች
		ባል የሞተባት
		የፈታች
11	የት/ት ደረጃዎት ምንድነው?	ምንም አልተማረኩም
		ከ 1-8 ተምሬያለሁ
		ከ 9-12 ተምሬያለሁ
		ዲፕሎማ እና ከዛ በላይ
12	ስራዎት ምንድነው?	የቤት እመቤት
		ገበሬ
		ሌላ የጉልበት ስራ

		በመንግስትና ድርጅት ቅጥር
13	ስያገቡ ዕድሜዎት ስንት ነበረ?	
14	በቅርብ ዘመድዎት እንደዚህ ዓይነት ህመም አለ ወይ?	አዎ
		አይደለም
	ለ. ከወሊድ ጋራ የቴያያዙ ምክንቶች	
15	ስንት ጊዜ ወልደዋል?	
16	ለመጨረሻ ጊዜ እንዴት ነበር የወለዱት?	ያለምንም ችግር በማህፀን በር በኩል
		በባለሙያች እገዛ በማህፀን በር በኩል
		በቀዶ-ጥገና
17	ለመጨረሻ ጊዜ ስወልዱ ዬት ነበረየወለዱት?	በጤና ተቀዋም
		ቤት
18	ቤት ወልደው ያውቃሉ?	አዎ
		አይደለም
19	ከወለዱ በኃላ ምን ያክል ቆይተው ስራ ይጀምራሉ?	42 ቀን በፊት
		42 ቀን በኃላ
20	ልጅዎችን ምን ያክል አራርቀው ይወልዳሉ?	
21	ለመጨረሻ ጊዜ ስወልዱ ምጡ ምን ያክል ቆይቶ ነበር?	
22		አዎ

	ምጥ ከ>24 ሰዓት በላይ ቆይቶበት ያውቃል?	አይደለም
23	ለመጀመሪያ ጊዜ ስወልዱ ዕድሜዎት ስንት ነበረ?	
24	መጨረሻ ጊዜ ስወልዱ ዕድሜዎት ስንት ነበረ?	
25	ስወልዱ ባለሙያ ውጫኛውን መውለጃ በር ቆርጠው/ ተቀደው ያውቃል?	አዎ
		አይደለም
26	ባለሙያ የተለየያ የማዋለጃ ወይንም ብረት በመጠቀም አዋልዶት ያውቀል?	አዎ
		አይደለም
ሐ. ውስጣዊ የሆድ ግፊትየሚጨምሩ መምክንቶች		
27	ከ 3 ሳምንት በልይ የቆየ ሳል ነበረዎት?	አዎ
		አይደለም
28	ከ 2 ወር በላይ የቆየ የሆድ ድርቀት ነበረዎት?	አዎ
		አይደለም
29	ከበድ ያለ ዕቃ ይሸከማሉ?	አዎ
		አይደለም

ተጠቂዎቹ ዘግይተው ለህክምና እንዲመጡ የሚያደርጉ ምክንያቶች

30	ህመሙ ከጀመሮት ስንት ጊዜ ሆነ?	
	**ለመታከም ለምን ቶሎ አልመጣሽም?**	
31	የሚደግፎትን አጥተው?	አዎ
		አይደለም
32	ገንዘብ በመጣት?	አዎ
		አይደለም
33	ትራንስፖርት በማጣት?	አዎ
		አይደለም
34	ህክምና የሚሰጡ ቦታዎች ስለራቁ?	አዎ
		አይደለም
35	ህመሙ በሴቶች ስለምከሰት ችግር የለውም ብለው?	አዎ
		አይደለም
36	ቀዶ ጥገና ስለምፈሩ?	አዎ
		አይደለም
37	በባህላችን አይፈቅድም?	አዎ
		አይደለም
38	ሰዎች ያገሉኛል/ይሸሹኛል ብለው ስለፈሩ?	አዎ
		አይደለም
39	ችግሮትን መግለጥ ስለፈሩ?	አዎ

		አይደለም
40	ስራ ስለምበዛበት ጊዜ ስላላገኙ?	አዎ
		አይደለም
