# Supplementary material for: Associated factors of pelvic organ prolapse among patients at Public Hospitals of Southern Ethiopia: A case-control study design
Source: PLoS One. 2023 Jan 18;18(1):e0278461. doi: 10.1371/journal.pone.0278461 (PMC9847956; doi:10.1371/journal.pone.0278461)
Supplement: Attachment — Submitted filename: Response to reviewers.docx [file pone.0278461.s002.docx]

**Response Letter**

**Authors:**

1. **Mr. Asfaw Borsamo**
2. **Mr. Mohammed Oumer**
3. **Mr. Ayanaw Worku**
4. **Mr. Yared Asmare**

# Editorial-in-Chief of the PLOS ONE JOURNAL

# May 21, 2022

**Dear Dr. (Professor) Adrian Stuart Wagg,**

Please find enclosed a revised manuscript entitled “**Predictors of pelvic organ prolapse among patients at Public Hospitals of Southern Ethiopia: A case-control study design’’** with the kind request to consider it for publication in the Plos One Journal.

In the first place, we would like to thank the Esteemed Dr. Adrian Stuart Wagg and Reviewers for your kind and lesson giving comments, create an opportunity to improve our work. We have accepted the comments, and we made modifications accordingly. For the concerns that were raised by the esteemed reviewers, we have given explanations.

**Responses to Reviewers:**

**Corrections/revisions for Reviewer 1**

**Abstract:**
1. The conclusion is largely valid but the last sentence is perhaps somewhat speculative. Perhaps the authors might like to modify this.

- Dear reviewer, we found this comment is relevant and we have corrected/revised as your request. Thank you very much for your suggestions and recommendations.

**Introduction:**
2. The authors note the highly variable prevalence of prolapse in studies which existed to date perhaps they might confine this detailed discussion to the discussion section and perhaps suggest some explanation as to why this is the case.

- Dear reviewer, the objective of the study is to determine predictors of the pelvic organ prolapse. This why we preferred to discuss in detail about the prevalence of the pelvic organ prolapse in introduction part.

3. The authors note established risk factors in other studies, could they therefore suggest why a study in Ethiopia would be of value or might produce different information?
Would a study attempting to intervene on the known modifiable factors be more significant and have greater utility than reiterating what is known? the authors make a good point about using the data with “responsible bodies” in playing a role of modification of such factors which would be a useful motivation upon which to build

- Dear reviewer, the most risk factors of the prolapse varies greater from the community to community because of different socioeconomic status, health system development level, and culture or beliefs. According to the literatures, so many factors (over 60) have association with the prolapse. Policy makers and health managers would not intervene all risk factors of the prolapse. This study was conducted with a case control study design to identify which factors are significantly affecting the prolapse in the study areas. Hence, managers and policy makers of the study areas possibly can work on identified factors to modify.

**Methods and materials**
4. It is good to see an accurate sample size calculation. what was the degree of imprecision in the prevalence estimate?

- Dear reviewer, previously, we had written the sample size calculation in short and summarized way. Now, on the revised manuscript, we have explicitly explained how the sample size was calculated as following:
- In this study, sample size was calculated by using Epi info version 7 software. For unmatched case-control study design, this software calculates by using double proportion formula [n= (p_1_q_1_ + p_2_q_2_) (f(α,β)) / ((p_1_ - p_2_)², n= number of sample size in each group, α = type I error, β = type II error]. To calculate sample size, we used proportions from the similar study conducted in Bahr Dar City, Ethiopia, as following: (see Table 1)

| Risk actors | % of case with exposure | % of control with exposure | Power | CI (%) | AOR | Sample size |
| --- | --- | --- | --- | --- | --- | --- |
| Age >44 | 71.8 | 27.1 | 80 | 95 | 6.8 | 55 |
| Formal education | 85 | 58 | 80 | 95 | 4.3 | 111 |
| Parity/delivery | 64.5 | 26.67 | 80 | 95 | 4.5 | 69 |
| Sphincter damage | 15.2 | 2 | 80 | 95 | 8 | 173 |
| Carry heavy object | 67.4 | 40 | 80 | 95 | 3.1 | 131 |
| Family history of POP | **22.6** | **6.2** | **80** | **95** | **4.9** | **176** |
| Underweight | 34.5 | 14 | 80 | 95 | 3.2 | 164 |
| Delivery assisted by non-health personnel | 63.4 | 39.1 | 80 | 95 | 2.6 | 167 |

- We got maximum sample size with variable of ‘family history of POP’ which is 173, after considering the design effect of two, and a 5% non-response rate, the total sample size became 369 patients (123 cases and 246 controls: in 1 to 2 cases to controls ratio).

5. Please give the authors describe their random sampling technique for participants and hospitals?

- Dear reviewer, among 14 governmental general and referral hospitals in SNNPR, seven hospitals were selected by simple random method (lottery method). All patients with a pelvic organ prolapse visiting gynecologic OPD during data collection period were included as cases. To select controls, we used systematic random sampling method, depending on gynecologic patient flow of each hospital. Hence, the K value for each hospital varies. For one prolapse case in each hospital we randomly included two controls. For example, when we had got 20 cases of prolapse in a gynecologic OPD of one hospital, we select 40 controls from the same OPD of that hospital by using systematic random sampling method

5. Was the Constipation question validated?

- Dear reviewer, we considered constipation lasting longer than three months as a significant enough to contribute for the development of the prolapse. Many other studies also agree with this statement.

6. Who examined the women?

- Gynecologists performed physical examination, history taking, and diagnosis as already explained in the manuscript.

7. The authors are to be congratulated in the conduct of this study in a challenging environment, in particular the governance processes they have put in place to ensure data quality.

The statistical analysis section is entirely appropriate.

- Dear reviewer, we thank you very much for understanding our efforts and challenging environment that we had faced.

8. I note that written informed consent was obtained from study participants, What proportion of women was illiterate and how was consent gained in this case?

- Dear reviewer, thank you for your important comments, a written informed consent was obtained directly from study participants who can read and write. For those unable to read and write, first the consent was clearly read to her and then her immediate/ significant family member (like husband or son, etc) will sign it. Even she has not family member, the consent was clearly read to her and then her fingerprints were taken as signature.

Results
9. For what reason have the authors included the religion of the women? Was this used as a controlling variable in parity? Did religion and parity interact?

- Dear reviewer, religion of the respondents first included as sociodemographic characteristic for the descriptive purpose. Later, its association with the prolapse checked on multivariable logistic regression and it did not show association. We have checked that no interaction of the religion and parity.

10. Is the proportion of women earning greater than 1200 birrs representative of the Ethiopian general population of women? Likewise, is the proportion having a home delivery typical?

- Dear reviewer, this study categorizes the income by using World Bank cut off point of poverty line, as operationally defined in the main manuscript. According to World Bank definition of the poverty line, earning below $1.25/day(1200 birr/month) is considered below poverty line. In our study, about 69% of the women with prolapse earn less than $1.25/day; about 17.47% of women from the control earn less than $1.25/day. In our study, 35.58% of the women with prolapse had at least one history of home delivery and about 30% of women from controls had at least one history of home delivery in their life. We don’t think shortage of the income affects home delivery in Ethiopian because medical services related with obstetrics is for free. Rather, home delivery is related to cultures, awareness, and attitude

11. Is the history of Sphincter damage and vagina tear when gained from the women reliable?

- Dear reviewer, yes, it is reliable because they never forget history that they had episiotomy or vagina tear. Moreover, their husbands had helped during the interview for some.

12. Regarding predictors, were any variables discarded on bivariate analysis? Were there any interactions? For example was high parity associated with relative poverty?

- Variables such as ethnicity, marital status, religion, residency, home delivery, duration of time to return to work after delivery, delivery interval, age at marriage, age at first delivery, age at last delivery, chronic cough and chronic constipation were discarded on bivariate logistic analysis.

13. Likewise was carrying heavy loads more common in women who were of lower educational attainment or low income?

- Dear reviewer, yes, carrying heavy loads more common in women who were of lower educational attainment or low income.

14. The results section is otherwise extremely well written But I should like to see some comparative analysis of the factors in table one in terms of prevalence of prolapse and say religion occupational status and ethnicity for completions sake.

- Dear reviewer, Table 2 shows these very clearly, and one can easily calculate from the table. We done in such way to reduce space and explain in summarized way..

15. Can the authors tell us what the average delay in seeking treatment was for the women both as a whole and according to income status education status or employment status?

This might help to support their argument that women with high income get better health services seek timely treatments and can hire someone to help them

- Dear reviewer, we found your concerns as very important and we have included it also in the main manuscript.
- The average delay of the women for the treatments since onset of the symptoms was 36.41 ± 3.94 months. Out of 123 women with the prolapse, 84.55% delayed for the treatments and only surgical interventions done as treatments. Among 104 women delayed for the treatment, 76% of them claimed lack of money as main reason for the delay. Out of 104 women delayed for the treatment, 67.48% (83) did not attend formal school and 69.1% (85) of them earn less than $1.25/ day.

Discussion

16. The authors present their findings in a logical manner and attempts to explain their findings.
    Might the authors also consider the amount of physical exertion women perform separate from carrying heavy loans as a potential mitigating factor - as the authors note this might also account for the higher prevalence of prolapse in underweight women.

- Of course, the reviewer is right, but for us it was difficult to measure amount of exertion force other than carrying heavy loads. We considered all type of heavy physical forces as carrying heavy loads.

17. The authors might consider comparing their sample with the general Ethiopian population to give the reader some frame of reference for example, were the women more educated, or likely to be employed or were they wealthier than average women in Ethiopia.

- Dear reviewer, we found this a very important comment and we incorporated this comment into the manuscript.

18. The high parity doesn't necessarily imply low utilization of family planning - the high parity may well be purposeful. do the authors have any evidence to suggest that women want fewer children?

- Dear reviewer, we appreciate you for rising important idea, and we have made modifications accordingly.
- In Ethiopia, there is low utilization of family planning especially among rural residents. This because women in rural Ethiopia are characterized by low education, low access to health informations, beliefs and cultures that encourages the women to have many children. In the study areas, having many children is considered as proud, dignity, and ultimate blessings from the Creator. Moreover, in the study areas, deciding the number of the children to have exclusively the right of the husbands, women have no right to decide.

19. The authors have considered their limitations and biases, notably the external validity of this hospital population seeking help versus the general population
    to what extent were husbands likely to give reliable information on their wives?

- During the data collection, we found that husbands’ helps were very important especially when women had forgotten some events in their life history. They knew almost everything about their wives.

20. In conclusion the authors have identified many potentially remedial factors in the development of prolapse. how likely are women to choose active treatment of their prolapse in Ethiopia.

- Dear reviewer, thank you for your comment and we included it into main manuscript accordingly.
- Most of the women arrived at hospital at advanced stage of the prolapse. They completely had hidden the problem and they had showed to their close relative when the condition got unbearable. As a result no options than surgical treatments. During the data collection, out of 123 women with prolapse 104 (84.55%) were surgically repaired (vaginal hysterectomies).

21. Is operative intervention financially out of reach for the majority of women?

- Dear reviewer, thank you for your comment and we included it into main manuscript accordingly
- Among 104 women delayed for the treatment, 76% of them claimed lack of money as main reason for the delay. Most of the women with prolapse come to hospital after they had got some financial support from some people who understood their conditions.

22. What conservative therapies are available? This information would certainly help add context to the paper

- Dear reviewer, rest of the women with less advanced stages ( stage II) were treated with conservative means of treatments like lifestyle and behavior modification, [physical therapy for the pelvic floor](https://www.voicesforpfd.org/about/physical-therapy/), and using [vaginal device (Pessary)](https://www.voicesforpfd.org/about/pessaries/)

**Corrections/revisions for Reviewer 2**

1. The authors investgated in the present study the effect estimate of several known factors that predispose to pop in an Ethiopian population. Despite their rigorous methodology i believe that the lack of an appropriate sample size (which is denoted by the large confidence intervals that are provided) is the main reason for rejection of this article.

- Dear reviewer, we thank you very much for your concern. The most risk factors of the prolapse vary greater from the community to community because of different socioeconomic status, health system development level, and culture or beliefs. According to the literatures, so many factors (over 60) have association with the prolapse. Policy makers and health managers would not intervene all risk factors of the prolapse. This study was conducted with a case control study design to measure which factors are significantly affecting the prolapse in the study areas. Hence, managers and policy makers of the study areas possibly can work on identified factors to modify.
- **Regarding sample size calculation,**
  - Dear reviewer, our sample size very high for case-control study and we calculated and got the value according scientific rule. Everything in this study is scientifically randomized.
  - Dear reviewer, previously, we had written the sample size calculation in short and summarized way. Now, on the revised manuscript, we have explicitly explained how the sample size was calculated as following:
  - In this study, sample size was calculated by using Epi info version 7 software. For unmatched case-control study design, this software calculates by using double proportion formula or power approach [n= (p_1_q_1_ + p_2_q_2_) (f(α,β)) / ((p_1_ - p_2_)², n= number of sample size in each group, α = type I error, β = type II error]. To calculate sample size, we used proportions from the similar study conducted in Bahr Dar City, Ethiopia, as following: (see Table 1)
  - We got maximum sample size with variable of ‘family history of POP’ which is 173, after considering the design effect of two, and a 5% non-response rate, the total sample size became 369 patients (123 cases and 246 controls: in 1 to 2 cases to controls ratio).

Table 1: sample size calculation

| **Risk actors** | **% of case with exposure** | **% of control with exposure** | **Power** | **CI (%)** | **AOR** | **Sample size** |
| --- | --- | --- | --- | --- | --- | --- |
| **Age >44** | 71.8 | 27.1 | 80 | 95 | 6.8 | 55 |
| **Formal education** | 85 | 58 | 80 | 95 | 4.3 | 111 |
| **Parity/delivery** | 64.5 | 26.67 | 80 | 95 | 4.5 | 69 |
| **Sphincter damage** | 15.2 | 2 | 80 | 95 | 8 | 173 |
| **Carry heavy object** | 67.4 | 40 | 80 | 95 | 3.1 | 131 |
| **Family history of POP** | **22.6** | **6.2** | **80** | **95** | **4.9** | **176** |
| **Underweight** | 34.5 | 14 | 80 | 95 | 3.2 | 164 |
| **Delivery assisted by non-health personnel** | 63.4 | 39.1 | 80 | 95 | 2.6 | 167 |
