# Supplementary material for: Associated factors of pelvic organ prolapse among patients at Public Hospitals of Southern Ethiopia: A case-control study design
Source: PLoS One. 2023 Jan 18;18(1):e0278461. doi: 10.1371/journal.pone.0278461 (PMC9847956; doi:10.1371/journal.pone.0278461)
Supplement: Attachment — Submitted filename: Response to Reviwers.docx [file pone.0278461.s003.docx]

**Response Letter**

**Authors:**

1. **Mr. Asfaw Borsamo**
2. **Mr. Mohammed Oumer**
3. **Mr. Ayanaw Worku**
4. **Mr. Yared Asmare**

# Editorial-in-Chief of the PLOS ONE JOURNAL

# October 03, 2022

**Dear Dr. (Professor) Adrian Stuart Wagg,**

Please find enclosed a revised manuscript entitled “**Associated factors of pelvic organ prolapse among patients at Public Hospitals of Southern Ethiopia: A case-control study design’’** with the kind request to consider it for publication in the Plos One Journal.

In the first place, we would like to thank the Esteemed Dr. Adrian Stuart Wagg and Reviewers for your kind and lesson giving comments, create an opportunity to improve our work. We have accepted the comments, and we made modifications accordingly. For the concerns that were raised by the esteemed reviewers, we have given explanations. We have checked that our references list is complete and correct.

**Journal requirement:** We have checked that our references list is complete and correct.

**Responses to Reviewers:**

**Corrections/revisions for Reviewer 1**

1. The authors have prepared an excellent response to the reviewers’ comments and have added in information which makes this an informative paper. The abstract might benefit from adding into the results the delay results – this would then justify their conclusion about awareness raising etc.

- Dear reviewer, we would like to thank for appreciating a previous response and we found the recent comment is also relevant. Hence, we have corrected/revised as your request. Thank you very much for your suggestions and recommendations. Your comments are making our work complete.

2. Id like to see the consent response added into the paper – this is useful for those conducting research in areas with high levels of illiteracy

- Dear reviewer, we used the consent form as following:

**Consent form**

***English version***

I have read or it was read to me (for those who cannot read and write) and understood well the condition stated above and I can withdraw from the study at any time and I understand that there is no risk of participating and no incentive to be given when I participate in the study. Therefore, I am willing to participate in the study. Signature___________________ Date____________

**Thank you so much!**

***Amharic version***

**የስምምነት ፍቃድ**

ከላይ የተጻፈውን መረጃ በደንብ አንብቤ ተረድቸው አለሁ፡፡ በጥናቱ ላይ መሳተፍ በኔም ሆነ በልጀ ላይ ምንም አይነት ችግር እንደማያደርስ ፤ የተለየ ጥቅማጥቅምም እንደለለው እንዲሁም በማነኛውም ሰአት ካልተመቸኝ የማቋረጥ መብት እንዳለኝ ተነግሮኛል፡፡ በመሆኑም በጥናቱ ላይ ለመሳተፍ ዝግጁ/ፍቃደኛ ነኝ ፡፡

ፊርማ --------------------- ቀን --------------------------------

ስለተሳትፎዎ እናመሰግን አለን !!!

**Corrections/revisions for Reviewer 2**

Thank you very much for your suggestions and recommendations.

1. If the predictive value of these factors is to be investigated a ROC and AUC analysis along with sensitivity and specificity of individual factors or the whole logistic regression should be provided. Otherwise the study should be targeted only around the risk factors and not their predictive role. If the predictive role is to be investigated the Hosmer Lemeshow goodness of fit of the logistic regression should be reported.

- Dear reviewer, thank you so much for your comment. We have modified our manuscript; we have replaced the term “*predictors*” with term “*associated factors*” throughout the manuscript. Mostly in public health research, researchers prefer the term “determinant” for cohort studies, “predictors” for case-control studies and retrospective cohort studies, and “associated factors” for cross-sectional studies. Similarly, we used the term “predictor” simply to show the level of term choice. So, thank you the Esteemed Academic Editor and Reviewer, now use the term “associated factors” Thank you very much for your suggestions and recommendations.

2. Despite having performed a sample size calculation, the confidence intervals that the authors report are ather wide; hence, an accompanying power calculation of their findings could help evaluate the actual robustness of their results and comment on them in the discussion.

- Dear reviewer, we have incorporated per your request in the discussion. Thank you very much.
